# Textile Supplies for Hospitals

**Published:** 1916-02-19

**Authors:** 


### TEXTILE SUPPLIES FOR HOSPITALS

### V. The Methods of Manufacture.

Textile manufacturing was a handicraft before it became a highly specialised mechanical industry, and the bare essentials of its practice cannot more clearly be illustrated than by reference to the primitive tools of the trade. The first essential was an instrument for reducing a chaotic mass of fibre to some definite order. A couple of short-handled rakes were used in preparing the long--fibred materials, and were called hackles or combs, accordingly as they were made for flax or for wool. Their function was to abstract the short and broken fibres and straighten the longer ones for the convenience of the spinster. The short wools and cotton, on the other hand, were scrambled into a wad by the aid of a pair of hand-cards, or square-backed brushes fitted with flexible wire bristles. More perfect and economical results are obtained upon the elaborate machinery that has been substituted for these rude appliances. The work is done upon rotary mechanism, but it is still in essence a process of combing, or of brushing, or of both, having for its object the production of a continuous sliver or ribbon of loosely connected fibre which in course of further manipulation can be attenuated and formed into yarn.

### Manual Processes.

This hand-prepared fibre was spun upon the domestic wheel, a simple contrivance for causing a spindle to revolve. The carded wool was attached to the spindle and as the wheel revolved the spinster drew the sliver finer, gave it a sufficiency of twist by rotating the wheel, and then wound up the length of spun yarn upon the spindle. What the spinster did by hand the spinning machine does by the action of speeded rollers, arranged to pay out prepared raw material at a faster rate than it is passed in, with the effect that the fibres are drawn one over another, and that the thread is reduced in thickness while the rotating spindle twists and winds up the completed thread.

The same duplication of a manual process takes place in weaving, but the warp or lengthwise threads are no longer stretched over a bough, nor is the shuttle thrown across them by hand. In fundamentals the textile industry does not alter with the ages, but in the detail of the practical application of ancient principles, few industries are more closely specialised. Where the spinster spun one thread and devoted to it the attention of her hand and eye, the frame or mule spins hundreds of threads simultaneously. The individual attention that was given to each bunch of fibre and foot of yarn is replaced by a sequence of operations each carrying the treatment one short stage forward, and designed to give in the long run a perfect uniformity to the whole. The business is made mysterious to the outsider by the employment of non-illuminative technical terms, and by the existence of endless incidental differences of practice, but a grip may always be kept upon the unchanged essentials of the case.

### Analogies in Machinery.

Linen is prepared upon a machine hackle, a ponderous machine in which down-hanging fringes of flax are combed by the passage through them of steel pins carried upon endless belts. Worsted wool, after being washed and dried mechanically and carded, is treated commonly on circular combs, rotating sharply in the horizontal plane and carrying minor circles also furnished with steel pins. Shorter worsted, some linen, and combed cotton are dealt with on machines suggested to the original inventor, Heilmann, by the sight of his daughter combing her hair. These machines reproduce faithfully the nip with the fingers and the passage of the comb through the held portion. Combing is in the nature of an extra process when applied to cotton, and combed yarn is distinguished from that which has been merely carded. The machine cards are cylindrical, with large drums furnished with elastic wire teeth to carry the raw material forward while it is worked on by smaller rollers, carrying not dissimilar teeth and rotating upon the circumference of the large cylinder. The teeth of the cards tease out the fibre into a mesh evenly laid across the width of the cylinder, and thence it is condensed again into a loose rope.

### Spindles and Looms.

The production of a thick sliver is the prelude to the preparation of successively finer and finer ones by drawing down the diameter gradually enough not to create weak or uneven places in the thread. In course of drawing, the material changes its name with its state, passing from "slubbing" by more or less delayed stages down to "roving" ready for the spinning machine. Cotton wefts and the yarns for tweeds, blankets, and flannels are all mule-spun, which signifies that they have been produced on a machine of intermittent action giving a particularly full and spongy yarn. The self-acting mule, one of the most fascinating pieces of machinery ever devised, repeats in close detail the manual movements of the domestic spinster. The whole front of the machine, carrying the spindles upon which the yarn is to be wound, runs forward, and while the roving is being stretched down to a precarious fineness, the spindles are at work throwing in the twist. The twist finds its way first into the weak places, strengthening them; and as the travelling front returns a stretched wire rises to support the newly-made yarn, and the spindles get to their work of winding the spun yarn upon the spindle. The mule, the invention of Arkwright, is the distinctive engine of the British cotton trade, and by weight of numbers the most important spinning machine of any.

Note.—Previous articles in this series appeared on December 18, 1915, and January 8, 22, and February 5, 1916. The next article will deal with "Hospital Blankets."

The ring-spun cotton that is used oftenest for warp is the product of a machine running at high speeds and requiring less skilled attention than the mule. The business of drafting the roving to the requisite fineness by roller action, the torsion of the thread, and its winding-on proceed simultaneously. The ring that gives the machine its name is a flanged steel ring, wide enough to encircle the spindle and bobbin, and around its flange works an ear-shaped "traveller" of bent wire under which the thread passes. The function of the traveller is to impose a light drag and facilitate continuous spinning at higher speeds than are practicable on spinning frames of some other types. The ring traveller replaces in fine-spinning the old-fashioned flyer, a leg of bent wire fitted with an eye and carried round by the rotation of the spinning-bobbins. Flyer-spun yarn, although technically perhaps the most perfect of any spun upon frame machines, is economically obtainable only in the coarser numbers.

Between spinning and weaving, yarn needs more or less rewinding, and such as is intended for warp has to be arranged in parallel threads of equal length. Warps liable to chafe in weaving are sized with mucilaginous matter and are then drawn into the loom. It is of the essence of weaving to obtain control of the warp threads so that alternate or selected threads may be lifted out of the plane of the remainder. This control is obtained through the heddle shafts, in which there are eyelets for the individual warp threads. As the loom works one shaft or another is lifted, carrying up with it the appropriate threads, and between the parted threads the weft is shot by the transit of the shuttle. The new thread is beaten home by an oscillating wire reed and weaving continues, the shafts lifting in their appointed order and the shuttle shooting back and forth at a speed which in weaving ordinary calicoes reaches 230 passages per minute. The speed is much lower in the wide looms employed for sheeting and in weaving woollens, or in producing fabrics with large floral patterns or lettering. Large fancy designs and woven lettering are obtained with the aid of the Jacquard, an apparatus controlled much as is the musical-box, and governing the lifts of the warp threads individually.

### Dyeing and Finishing.

Weaving supplies a thread structure, but few fabrics pass into consumption precisely in the state in which they leave the loom. They undergo processes of finishing whether for the bare sake of appearances or with some more profound motive. At the minimum the process of finishing is akin to laundering, a simple washing and ironing, and at the maximum the goods are transformed out of all recognition by treatments as elaborate as those by which the cloths are made. Woollens on leaving the loom are oily with the oils used as lubricant for the fibre, and these require to be removed. Then woollens and worsteds are normally woven a good deal wider than their finished width to allow for the contraction that takes place upon wetting and for the effect of operations that are intended to consolidate the cloth. Cottons, even when they are to be used dyed or printed, have first to be bleached, and the act of bleaching involves some six or eight separate and severe processes. Cloths have to be strained out to uniformity in width; to smarten them, the down of projecting fibre upon their surface has to be removed by singeing or by shearing; or again, where a fluffy surface is wanted in the interests of warmth, the fibre has to be scratched up to form a pile.

Dyeing is, or may be, done at any of the three principal stages, and the most perfect penetration is obtained by dyeing fibre in the loose. Yarns are dyed in some circumstances after spinning, but the great preponderance of plain goods are dyed in the woven piece. Science and invention have brought vacuum dyeing vessels into use to economise steam and dye liquor, but the major improvements in dyeing relate to the colours in use. The coal-tar colours have been erroneously accused of fugitiveness and crudity, whereas in fact the best of them are the most stable colours that can be employed. It is, however, mainly because their use is simple, cheap, and certain that anilines ousted the vegetable colouring matters that have in part regained their kingdom during the war. Few colours, and those ones good, are wanted in textiles for hospital use—indigo or alizarin blue, Turkey red, and sulphur black are the ones generally to be recommended.

The results rather than the details of manufacture are the main matter from the consumer's point of view, for it is with the results that they have to deal. While in a particular case there are always incidentals which do not lend themselves to verbal description and which require concrete illustration in the form of a sample of the cloth, there are points that can definitely be fixed and can be used in the specifications upon which manufacturers are invited to tender. Indeed, there can be no real standardisation apart from definite particulars laid down. These details can best be formulated from the ascertained facts about articles of recognised suitability for their purpose. Some knowledge of how textile goods come into being is valuable in indirect ways, and of assistance in arriving at the fixed points at which one must aim in getting the best value for money. The possible has to be distinguished from the impossible, the natural limitations and the available alternatives have to be recognised, and these considerations form the excuse for the foregoing general survey, leading up to a more particular application of the principles involved. Given fabrics reflect the materials from which they have been made, and the processes through which they have been put, and the succeeding articles, dealing with specific goods one by one, will aim at definition in the case of each. The alternative routes by which one and the same objective may be reached may be left to the manufacturer, providing always that the main end is served. It appears, however, that there is room for explanation of what some of these ends should be and of the several varieties of goods which in one measure or another meet them.

